# The Potential of Self‐Maintainable Living Materials

**DOI:** 10.1002/advs.78082

**Published:** 2026-10-14

**Authors:** Mohammadsadegh Nadimifar, Tatiane Eufrásio‐da‐Silva, Lidia Maeso, Gorka Orive, Alireza Dolatshahi‐Pirouz

**Affiliations:** ^1^ Department of Health Technology Technical University of Denmark Kongens Lyngby Denmark; ^2^ Basque Sustainable Pharmacy & Biotherapy Research Group School of Pharmacy University of the Basque Country (UPV/EHU) Vitoria‐Gasteiz Spain; ^3^ Bioaraba Health Research Institute Vitoria‐Gasteiz Spain; ^4^ Regenerative Medicine BTI Biotechnology Institute Vitoria‐Gasteiz Spain

**Keywords:** autonomous materials, biohybrid scaffolds, closed‐loop feedback regulation, engineered living materials, self‐maintainable systems, stimuli‐responsive biomaterials, synthetic biology

## Abstract

Engineered living materials (ELMs) are emerging as a new class of autonomous and self‐maintainable systems that integrate metabolically active organisms within structural scaffolds to create materials capable of sensing, self‐repair, adaptive response, and long‐term functional persistence. Unlike conventional smart materials, which remain externally programmed and limited in regenerative capacity, ELMs leverage the intrinsic capabilities of microorganisms, fungi, algae, and mammalian cells to support continuous environmental interaction and feedback‐regulated behavior. These systems retain metabolically active biological components that can grow, sense, communicate, adapt, and, in some cases, self‐repair and self‐regenerate, positioning autonomy as the defining capacity of these next‐generation materials. Recent advances in synthetic biology, including programmable gene circuits, quorum‐sensing communication, and metabolic engineering, enable living components to execute closed‐loop decision‐making and maintain functionality with minimal external input. These capabilities are driving innovations across construction, agriculture, environmental remediation, bioenergy, and biomedicine, where ELMs enable functions such as damage‐triggered mineralization, adaptive pollutant degradation, and therapeutic self‐regulation. Despite this promise, key challenges remain in evolutionary stability, biocontainment, long‐term viability, and scalable manufacturing. This Review examines the design principles, current challenges, and translational pathways toward self‐maintainable and autonomous living materials capable of sustained operation in real‐world environments.

## Graphical abstract

Self‐maintainable living materials are presented as the next generation of bioadaptive systems, integrating continuous sensing, decision‐making, response, self‐repair, and adaptation in a closed‐loop framework. This review highlights the design principles and emerging technologies that enable autonomous, long‐term functionality for future regenerative and therapeutic applications.

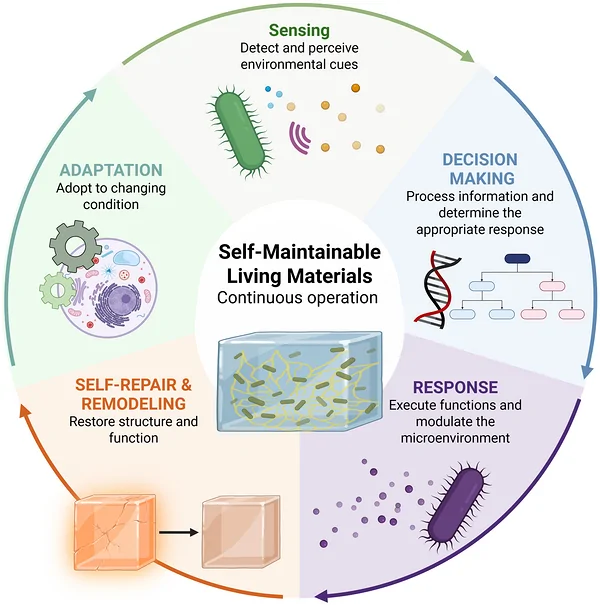

## 1 Living Materials: A Paradigm‐Shifting Venture Founded in the Union of Synthetic Biology and Materials Science

Materials science has evolved from the development of passive structural materials toward increasingly dynamic and responsive systems. Over recent decades, advances in biodegradable materials, biomimetic materials, and smart materials have transformed the field by enabling materials to interact with their surroundings and respond to external stimuli such as temperature, humidity, pH, light, pressure, magnetic fields, and electrical signals [1, 2, 3]. Smart materials, including shape‐memory materials, self‐healing polymers, piezoelectric systems, and magnetostrictive materials, have demonstrated the ability to reversibly alter their properties, recover functionality after damage, and adapt to changing environmental conditions [4]. Despite these advances, most smart materials remain predominantly abiotic systems whose responses are largely predefined by their design and are often limited in their capacity for adaptation, regeneration, and long‐term autonomous function [4]. Inspired by the adaptability, self‐organization, and resilience of biological systems, the next generation of advanced materials increasingly seeks to integrate living organisms directly into engineered material frameworks [5]. This paradigm has led to the emergence of engineered living materials (ELMs), a new class of biohybrid systems that combine metabolically active organisms with material architectures to enable functions such as sensing, growth, self‐maintenance, self‐repair, adaptation, and biological feedback regulation [5, 6].

ELMs are generally defined as engineered materials composed of living cells that either form or assemble the material itself and actively modulate its functional performance after fabrication [5, 6]. Unlike traditional biomaterials or smart materials, ELMs retain metabolically active biological components that can grow, sense, communicate, adapt, and, in some cases, self‐repair and self‐regenerate [7, 8]. Through the integration of genetically programmable living systems with structural matrices, ELMs blur the conventional boundary between living organisms and synthetic matter. By incorporating metabolically active components capable of continuous environmental interaction, ELMs represent a transition from responsive materials toward increasingly autonomous material systems [9].

The conceptual foundation of ELMs originates from the convergence of two rapidly evolving disciplines. Synthetic biology provides design‐build‐test‐learn strategies for programming living cells with user‐defined functionalities, while classical materials science contributes process‐structure‐property relationships that enable the fabrication of robust and functional materials [6, 10]. The fusion of these disciplines has enabled researchers to engineer microorganisms, fungi, algae, mammalian cells, and multicellular consortia capable of producing biologically active materials with programmable structural, chemical, electrical, and mechanical properties. In these systems, the nonliving matrix provides mechanical stability, environmental protection, and spatial organization, while the living component contributes biosynthesis, sensing, environmental responsiveness, self‐healing, and adaptive functionality [11].

Recent advances in genetic engineering, CRISPR technologies, metabolic engineering, and programmable synthetic gene circuits have significantly accelerated the development of ELMs [12, 13]. These technologies enable precise regulation of cellular behaviors such as quorum sensing, biofilm formation, metabolite production, and environmental responsiveness [14]. In particular, quorum‐sensing‐mediated communication has emerged as a critical mechanism enabling collective cellular coordination within living materials. By integrating quorum‐sensing‐responsive systems into material design, researchers have begun developing living smart interfaces capable of dynamically sensing microbial activity, responding to pathogenic signals, releasing therapeutic agents, and modulating local material properties in real time [15]. These developments represent an important transition from passive stimuli‐responsive materials toward biologically interactive systems capable of autonomous feedback regulation.

The potential applications of ELMs now extend across numerous scientific and technological domains, including biomedicine, biosensing, soft robotics, environmental remediation, sustainable construction, wearable electronics, energy systems, and adaptive infrastructure (Figure 1) [16, 17]. In biomedical systems, living materials have demonstrated capabilities for programmable drug delivery, infection‐responsive therapeutic release, tissue regeneration, wound healing, and immune modulation [15]. In environmental and industrial contexts, ELMs have been engineered for pollutant degradation, carbon sequestration, biosensing, and production of high‐value biomolecules [17, 18]. Living composite materials and bioinspired structural systems are also increasingly explored as sustainable alternatives to conventional materials due to their low‐energy biosynthesis, renewable production pathways, and potential for growth and self‐repair [12].

**FIGURE 1:**
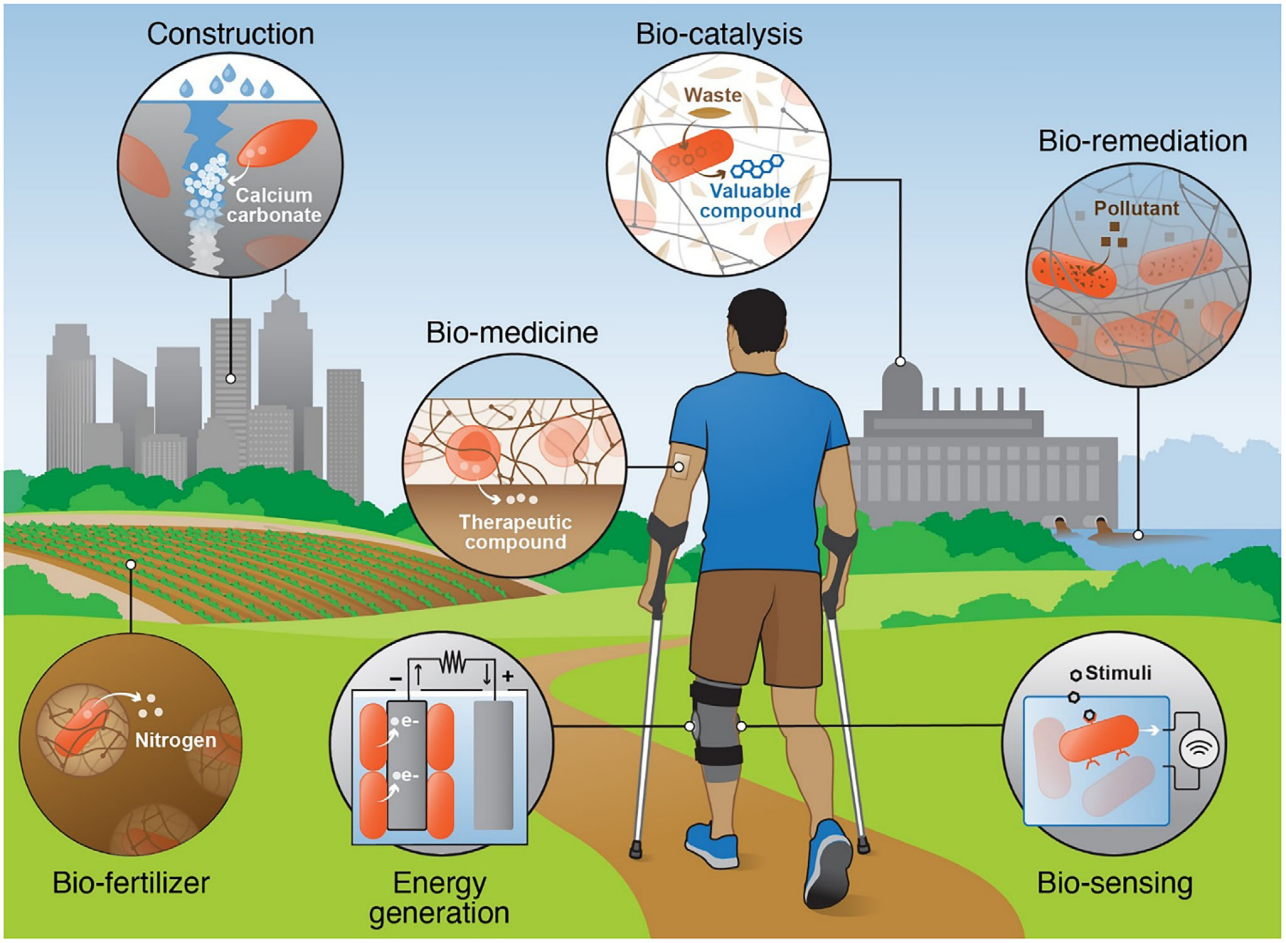
Different applications of living materials. Integrating cells or microorganisms into materials could transform various fields, such as construction, biocatalysis, biofertilization, bioremediation, energy production, biosensing, and biomedicine.

Despite the extraordinary promise of ELMs, several major scientific and translational challenges remain unresolved. Maintaining long‐term cellular viability, ensuring reliable biocontainment, achieving scalable manufacturing, balancing biological activity with mechanical robustness, establishing robust closed‐loop feedback regulation capable of continuously sensing and adapting to environmental changes, and controlling long‐term evolutionary stability remain significant barriers for practical deployment. Furthermore, regulatory concerns regarding biosafety, environmental release, horizontal gene transfer, and ethical management of programmable living systems continue to require careful consideration. The integration of multiple organisms into cooperative microbial consortia, the development of standardized metrology frameworks, and the improvement of long‐term functional persistence under real‐world conditions are also critical future directions for the field [5, 6, 17, 19].

This Review examines recent advances in ELMs across major application domains, with particular emphasis on their progression from responsive living materials toward self‐maintainable and increasingly autonomous systems. We compare the biological and material design strategies that enable sustained functionality, adaptation, and feedback regulation, while identifying the major barriers to real‐world translation. Finally, we discuss the technological and regulatory advances needed to develop reliable and autonomous living material systems.

## 2 Application Domains of Living Materials

The five application domains examined below illustrate how the same core design logic of pairing living cells with engineered matrices plays out very differently depending on the biological organism, the scaffold architecture, and the problem being solved. Table 1 provides a comparative summary of the living components, scaffold materials, functional mechanisms, and principal translational barriers in each domain.

### 2.1 Construction and Infrastructure

Concrete has been the dominant construction material for the past 200 years; however, its susceptibility to cracking and deterioration has motivated the development of living self‐healing approaches [20]. One strategy involves embedding bacteria capable of precipitating calcium carbonate (CaCO_3_), which can seal cracks following activation by moisture and oxygen [21, 22]. Different microbial pathways, including ureolysis, photosynthesis, denitrification, and sulfate reduction, can contribute to carbonate formation, with alkaliphilic bacteria being particularly suitable for the high‐pH concrete environment. Laboratory studies have reported recovery of compressive strength by up to 60% after 28 days of bacterial healing [23]. For example, Su et al. encapsulated *Bacillus alcalophilus* spores and nutrients within expanded vermiculite particles protected by a low‐alkali cement shell. This approach preserved bacterial viability within the concrete and enabled microbially induced calcium carbonate precipitation after water infiltration, resulting in nearly complete healing of cracks up to 0.5 mm after 28 days [24].

Beyond bacteria‐mediated self‐healing concrete, construction‐oriented ELMs employ diverse biological strategies, including fungal mycelium composites, microbial biocementation systems, algae‐integrated façade technologies, and regenerative architectural materials, each addressing different structural and environmental challenges [21, 25]. What distinguishes these systems from conventional building materials is not merely the presence of cells, but their capacity for metabolically driven behavior: growth, mineral deposition, gas exchange, and damage response that no synthetic material can replicate once deployed [26]. Fungal mycelium composites, for instance, self‐assemble into lightweight structures using lignocellulosic substrates as nutrient sources and can partially restore structural continuity after local damage under favorable conditions. Depending on species selection and post‐processing, these materials may achieve mechanical properties comparable to some synthetic insulation foams while remaining fully biodegradable. However, limited structural strength, moisture sensitivity, and batch‐to‐batch variability during biological growth continue to restrict their use in load‐bearing applications [6, 27].

Photosynthetic living materials also represent an emerging direction in sustainable construction [17]. Algae‐integrated façade systems and hydrogel‐supported photobioreactors have been developed to combine structural functionality with carbon capture, biomass production, and environmental regulation. In these systems, photosynthetic microorganisms can dynamically modulate carbon dioxide uptake and light absorption while simultaneously contributing to thermal regulation within buildings. Such living façade technologies may improve energy efficiency and reduce urban environmental impact by coupling biological activity with architectural design [28, 29]. Nevertheless, several engineering challenges remain unresolved, including maintaining adequate nutrient and light transport, preventing contamination and biofouling, and ensuring stable cellular activity during long‐term outdoor operation [6, 26, 30].

**TABLE 1:** Comparative overview of engineered living materials across major application domains.

Application area		Biological component	Scaffold / Matrix	Functional mechanism	Transitional limitation	Ref
Construction & Infrastructure	Mineralized systems	Ureolytic bacteria (Bacillus spp., cyanobacteria)	Concrete matrix—cementitious materials	Damage‐triggered CaCO_3_ biomineralization for crack sealing and structural repair	Limited microbial viability in alkaline environments, uneven activation, and scalability challenges	[23, 31, 32]
	Biofabricated composites	Fungal mycelium	Lignocellulosic composite substrates	Biological self‐assembly and regenerative lightweight structural formation	Low mechanical strength, moisture sensitivity, and variability during biological growth	[33]
	Environmentally responsive architectural systems	Microalgae—cyanobacteria	Hydrogel‐supported photobioreactors and façade systems	Carbon sequestration, thermal regulation, and dynamic environmental interaction	Biofouling, nutrient transport limitations, and long‐term outdoor operational stability	[34]
Agriculture and Food Systems	Agricultural living materials	Plant growth‐promoting rhizobacteria, and photosynthetic (microalgae, cyanobacteria)	Hydrogels (e. g., Alginate/carboxymethyl chitosan) and biohybrid hydrogel‐based living matrices	Controlled microbial delivery, root colonization, nutrient cycling, carbon sequestration, and adaptive metabolic regulation within agricultural environments	Environmental variability, competition with native microbiota, light and nutrient transport limitations, biofouling, and maintaining long‐term microbial stability under field conditions	[35, 36, 37]
	Living biocatalytic food and bioprocessing systems	Genetically engineered microorganisms (e.g., *E. coli, S. elongatus*, *M. guilliermondii*)	Hydrogel‐based biohybrid matrices and immobilization platforms	Metabolic conversion of low‐value feedstocks into high‐value food precursors, bioactive compounds, and industrial biomolecules	Metabolic burden, diffusion limitations, genetic instability, process scalability, and biocontainment challenges	[18, 38]
Environmental Remediation	Sequestration‐based systems	Fungi, bacteria, and microalgae	Hydrogel‐based matrices and biofilm scaffolds	Biosorption, bioaccumulation, and detoxification of heavy metals and environmental contaminants	Limited adsorption capacity, regeneration inefficiency, environmental instability, and reduced long‐term microbial viability	[35, 39]
	Biodegradation‐based systems	microalgae (e.g., *Chlorella zofingiensis*) and pollutant‐degrading bacteria	Hydrogels and biohybrid matrices	Enzymatic degradation of toxic organic pollutants, reusable contaminant removal, and enhanced stress tolerance	Diffusion limitations, microbial leakage, hydrogel fouling, scalability challenges, and maintaining remediation efficiency under fluctuating environmental conditions	[40]
	Photosynthetic carbon‐sequestering systems	Cyanobacteria and microalgae	Hydrogel‐based biohybrid scaffolds, photobioreactors, and living photosynthetic matrices	Photosynthetically driven CO_2_ capture, nutrient cycling, oxygen production, and environmentally responsive metabolic regulation	Light penetration limitations, biofouling, nutrient transport constraints, contamination risk, and outdoor operational instability	[17, 41]
Bioenergy System	Biocatalytic Energy Conversion	Engineered bacteria, yeast, algae, and microbial consortia	Hydrogels, polymeric matrices, conductive carbon scaffolds	Metabolic conversion of biomass or organic substrates into biofuels such as hydrogen, ethanol, methane, and bioelectric intermediates	Metabolic burden, low conversion efficiency, limited long‐term stability, substrate diffusion limitations, scalability of bioprocesses	[42]
	Microbial Fuel Cells (MFCs)	Electroactive bacteria such as Geobacter and Shewanella	Conductive electrodes, nanocomposite‐coated anodes, carbon felt, conductive hydrogels	Direct conversion of organic matter into electricity through extracellular electron transfer (EET); simultaneous wastewater treatment and energy recovery	Low power density, electron‐transfer inefficiency, electrode fouling, microbial competition, scalability and commercialization challenges	[43, 44, 45]
	Photosynthetic Bioenergy	Cyanobacteria and microalgae	Alginate hydrogels, bioprinted living matrices, semiconductive biohybrid scaffolds	Light‐driven energy harvesting, CO_2_ fixation, bioelectricity generation, and solar‐to‐chemical energy conversion	Dependence on illumination conditions, limited photosynthetic efficiency, oxygen sensitivity, long‐term viability, and environmental fluctuations	[41, 42]
Biomedicine and Wearable Systems	Therapeutic Living Materials	Engineered probiotics and bacteria (e.g., E. coli, Bacillus), viruses, stem cells, immune cells, Transfected mammalian cells	Injectable hydrogels, encapsulation matrices, natural/biological‐derived scaffolds, synthetic/hybrid based scaffolds, polymeric biointerfaces, pharmaceutical delivery systems	Targeted drug delivery, immunomodulation, regenerative therapy, wound healing, localized therapeutic secretion	Host immune activation, LPS‐induced inflammation, bacterial translocation, genetic instability, long‐term containment, microbiome imbalance, and biosafety concerns	[46, 47]
	Biosensing and Diagnostic Living Materials	Engineered bacteria, microbial biosensors, living cells with synthetic genetic circuit	Responsive hydrogels and systems, biohybrid sensing matrices, functionalized materials, diagnostic systems	Real‐time biomarker detection, protease sensing, DNA sensing, inflammation monitoring, programmable signal transduction	Signal instability, false activation, limited in vivo robustness, biocontainment challenges, immune‐mediated interference, sensor drift during long‐term operation	[48, 49, 50]
	Wearable and Bioelectronic Living Systems	Conductive bacterial biofilms, electrogenic bacteria, enzymes, living bioelectronic interfaces	Conductive hydrogels, PEDOT:PSS interfaces, wearable biofuel cells, flexible electronics, BioMEMS platforms	Bioenergy harvesting, physiological monitoring, self‐powered biosensing, adaptive bioelectronic communication	Bioelectronic instability, moisture sensitivity, limited long‐term durability, inflammatory tissue responses, electrode‐biofilm degradation, and mechanical mismatch with tissues	[48, 51]
	Tissue‐Integrated and Adaptive Biointerfaces	Mammalian cells, organoids, neural cells, engineered bacteria, electrogenic living systems	Biointegrated hydrogels, microfluidic systems, self‐healing conductive matrices, adaptive ECM‐inspired scaffolds	Long‐term tissue integration, regenerative interfacing, neural communication, dynamic tissue remodeling, self‐healing biointerfaces	Chronic immune activation, fibrosis, foreign‐body response, vascularization limitations, interface degradation, microbial persistence control, long‐term implantation safety	[16, 52]

Taken together, these examples illustrate that, despite targeting different construction challenges, successful ELMs rely on the same fundamental design principle: integrating living organisms as active functional components rather than passive additives. The critical unresolved questions are not conceptual but practical. How will adequate microbial viability be maintained in the alkaline chemistry of concrete? How will uniform bacterial distribution be ensured at the scale of a structural slab? How will long‐term performance be predicted when the biological component continues to evolve? Future progress will require synthetic biology tools that improve cellular robustness under construction‐specific stressors, computational models capable of predicting crack‐healing kinetics, and standardized testing protocols that regulators and engineers can truly use.

### 2.2 Agriculture and Food Systems

The central challenge in agricultural and food applications of ELMs is not fabrication but field performance: microorganisms that function reliably in controlled laboratory conditions frequently fail when confronted with soil pH variability, competing native microbiota, desiccation, and fluctuating temperatures [53, 54]. The engineering response has been to treat the scaffold not as a passive carrier but as a microenvironment that actively buffers these stressors. Hydrogel matrices used in current systems regulate nutrient diffusion, water retention, oxygen transport, and local pH, thereby extending microbial viability and preserving metabolic phenotype under conditions that would otherwise suppress biological activity [19].

In agricultural applications, ELMs have emerged as promising platforms for enhancing nutrient cycling, microbial stabilization, and localized plant–microbe interactions [55, 56]. Living fertilizers have been developed by embedding soil‐beneficial microorganisms, such as Rhizobium and Azospirillum, into peat, alginate, or hydrogel‐based matrices to enable controlled microbial delivery and improve bacterial survival in soil environments [57, 58, 59]. For example, encapsulation of plant growth‐promoting rhizobacteria within carboxymethyl chitosan and sodium alginate hydrogels increased bacterial colonization of plant roots, improved microbial viability in acidic soil conditions, and enhanced crop yield by approximately 30% [35]. Encapsulation within hydrogel matrices further protects rhizobacteria against desiccation, oxidative stress, and pH fluctuations while enabling localized rhizosphere metabolic activity and nutrient exchange [57, 59]. Emerging photosynthetic living materials based on microalgae and cyanobacteria further expand the agricultural potential of ELMs by enabling nutrient cycling, carbon sequestration, and environmentally responsive metabolic regulation. Environmental variables such as hydration, illumination, gas diffusion, and nutrient transport can dynamically influence microbial phenotype, metabolic activity, and long‐term functionality within these living agricultural materials [34]. Recent advances in engineered plant living materials further suggest the possibility of constructing genetically programmable agricultural systems capable of spatial organization, adaptive metabolic functionality, and environmentally responsive behaviors through 3D bioprinting and engineered hydrogel scaffolds [60].

Living materials have also gained increasing attention in food production and bioprocessing through the development of metabolically programmable biocatalytic systems. In these platforms, immobilized microorganisms are utilized to convert low‐value agricultural feedstocks, such as lignocellulosic biomass and starch‐rich waste streams, into high‐value compounds like amino acids, vitamins, flavors, and bioactive metabolites. Along these lines, a living material composed of genetically engineered *Escherichia coli* embedded within a polyvinyl acetate (PVA) hydrogel has been developed for the biosynthesis of valuable flavonoids such as naringenin and pinocembrin from coumaric and cinnamic acid precursors [18]. In such systems, the hydrogel matrix not only provides physical containment of engineered microorganisms but also regulates substrate diffusion, microbial proliferation, and metabolic exchange, thereby enabling sustained and controllable biosynthesis [18]. Recent advances in coculture‐based ELMs further suggest that spatial compartmentalization and microenvironment regulation can improve metabolic division of labor and stabilize complex microbial biomanufacturing systems [38]. These strategies may ultimately support broader applications in sustainable chemical manufacturing and industrial biotechnology. Although agricultural and biomanufacturing ELMs rely on similar principles of microbial stabilization and controlled mass transport, their practical constraints differ considerably: agricultural systems must function under highly variable field conditions, whereas biomanufacturing systems operate in more controlled environments where process stability and productivity can be more tightly regulated.

The core gap between current performance and field deployment is specificity to local conditions. Laboratory demonstrations of rhizosphere ELMs typically use homogeneous soils, controlled irrigation, monoculture settings, and standardized application methods that bear little resemblance to the diversity of soil conditions, microbial communities, and delivery practices encountered in real agricultural environments. The most pressing research needs are therefore not further proof‐of‐concept studies but systematic work on microbial competition dynamics in native soil communities, genetic stability over multiple growing seasons, and scalable manufacturing approaches that can deliver living materials at agronomically relevant volumes without compromising biological viability during transport and storage.

### 2.3 Environmental Remediation

Environmental remediation has emerged as one of the most promising application domains for ELMs due to their capacity for dynamic interaction with pollutants, environmental stressors, and ecosystem‐level processes through biologically mediated remediation pathways. The choice of living component is closely linked to the target pollutant and the required remediation mechanism. Cyanobacteria, algae, and microbial systems can remove or immobilize heavy metals through biosorption, bioaccumulation, and related mechanisms, whereas photosynthetic algae and cyanobacteria can additionally contribute to CO_2_ capture and carbon sequestration [39, 61, 62, 63, 64]. More broadly, environmental ELMs can therefore be viewed as operating through several main design strategies, including pollutant capture or immobilization, biological degradation or transformation, and photosynthetically driven carbon fixation [17, 65]. Recent advances in ELMs further suggest that the integration of biological functionality with programmable material architectures may enable remediation systems capable of dynamically responding to environmental fluctuations and contaminant stress [19].

Entrapment of microorganisms within hydrogel matrices, biofilms, and biohybrid scaffolds can substantially improve remediation efficiency by enhancing microbial stability, stress tolerance, and long‐term metabolic activity under variable field conditions. Beyond simple immobilization, these matrices regulate nutrient transport, pollutant diffusion, hydration, oxygen exchange, and localized physicochemical stability, preserving microbial viability and metabolic performance. The scaffold also functions as a protective microenvironment, shielding embedded organisms against oxidative stress, toxic contaminant exposure, and mechanical instability while enabling controlled pollutant‐cell interaction. Biofilm‐based systems may further benefit from quorum‐sensing‐mediated coordination, improving degradation efficiency and resilience under harsh conditions [66, 67]. Recent advances in biofilm‐based ELMs further suggest that multicellular metabolic coordination can significantly enhance both degradation efficiency and environmental resilience [56].

For example, *Chlorella zofingiensis* microalgae have been encapsulated within bioprinted double‐network hydrogels composed of poly(ethylene glycol) diacrylate (PEGDA) and sodium alginate coated with tannic acid to generate retrievable living materials capable of degrading tetracycline antibiotics with efficiencies exceeding 99%. This biohybrid system demonstrated enhanced mechanical stability, prolonged cellular viability, and minimal microbial leakage below 1.3% over seven days while maintaining degradation capability even at tetracycline concentrations as high as 400 mg/L. Importantly, the hydrogel matrix protected the microalgae against environmental stress and mitigated several limitations associated with conventional suspended microalgal systems, including uncontrolled proliferation and reduced tolerance to pharmaceutical pollutants [40]. The retrievability and reusability of these living materials further highlight the potential of integrating biologically active microalgae with mechanically robust scaffolds for sustainable water‐treatment applications. From a translational perspective, retrievable and immobilized ELMs may be particularly suitable for on‐site water treatment because they facilitate material recovery and reduce the risk of uncontrolled release compared with freely dispersed microorganisms [68, 69]. These systems demonstrate how biologically interactive matrices can support environmentally responsive remediation platforms capable of maintaining microbial metabolic activity under high contaminant stress while enabling controllable and reusable pollutant degradation. However, remediation efficiency in such systems remains strongly influenced by environmental variables including illumination, nutrient availability, pollutant heterogeneity, hydraulic conditions, temperature fluctuations, and long‐term microbial stability [17].

Beyond pollutant degradation, photosynthetic living materials based on cyanobacteria and microalgae are increasingly being explored as adaptive environmental systems capable of continuous CO_2_ sequestration, oxygen production, and nutrient cycling [63, 70]. These systems rely on dynamic metabolic activity and environmentally responsive functionality, allowing remediation performance to adapt continuously to changes in nutrient availability and gas‐exchange conditions. In photosynthetic living materials, integrating carbon sequestration with biohybrid architectures offers continuous biological activity, although practical performance remains strongly dependent on light availability, nutrient supply, gas transport, and long‐term cellular stability [71, 72]. In parallel, engineered microbial consortia and synthetic biology‐enabled living materials are emerging as promising strategies for developing autonomous remediation systems capable of feedback‐regulated pollutant sensing, adaptive metabolic activation, and self‐regulated environmental responses through engineered genetic circuits and multicellular cooperation [56]. Emerging studies further suggest that integration of biosensor‐assisted monitoring, synthetic regulatory circuits, and AI‐guided environmental control may enable future remediation systems with enhanced adaptive regulation, long‐term operational stability, and partially autonomous environmental responsiveness [17].

The field's practical ceiling is currently set by two problems that are specific to remediation contexts and rarely acknowledged in laboratory reports. First, contaminant heterogeneity: real polluted sites contain mixtures of heavy metals, organic compounds, and microplastics at variable concentrations, yet most ELM demonstrations target a single, well‐characterized pollutant under controlled conditions. Consequently, many current systems should still be considered laboratory‐scale demonstrations rather than field‐ready remediation technologies, particularly where long‐term performance, recovery of the living material, and biological containment have not been evaluated under realistic environmental conditions. Second, ecological risk: releasing engineered organisms into open environments, even within scaffolds, raises containment questions that standard biosafety frameworks were not designed to address. Therefore, advancing living remediation systems toward field deployment will require both technical progress in long‐term microbial stability, scalable biomanufacturing, and process control under heterogeneous conditions and parallel development of regulatory frameworks capable of evaluating biological risk at ecosystem scale [17].

### 2.4 Bioenergy Systems

Among the diverse applications of ELMs, bioenergy platforms are a highly attractive approach for sustainable energy generation and storage. These living systems utilize the metabolic activity of microorganisms and photosynthetic organisms to convert organic matter, waste streams, and solar energy into electrical or chemical energy. Broadly, these systems follow two complementary strategies: microbial electrochemical platforms that convert organic substrates into electrical energy and photosynthetic living systems that use light and CO_2_ to generate bioelectricity or energy‐rich products. Due to their environmental responsiveness, self‐regenerative capacity, and adaptive functionality, these systems are being explored as sustainable solutions for decentralized energy production and waste‐to‐energy conversion [42, 73]. Advances in synthetic biology and materials science have further enabled the development of engineered living energy materials with programmable and environmentally responsive behaviors (Figure 2A) [19, 56].

**FIGURE 2:**
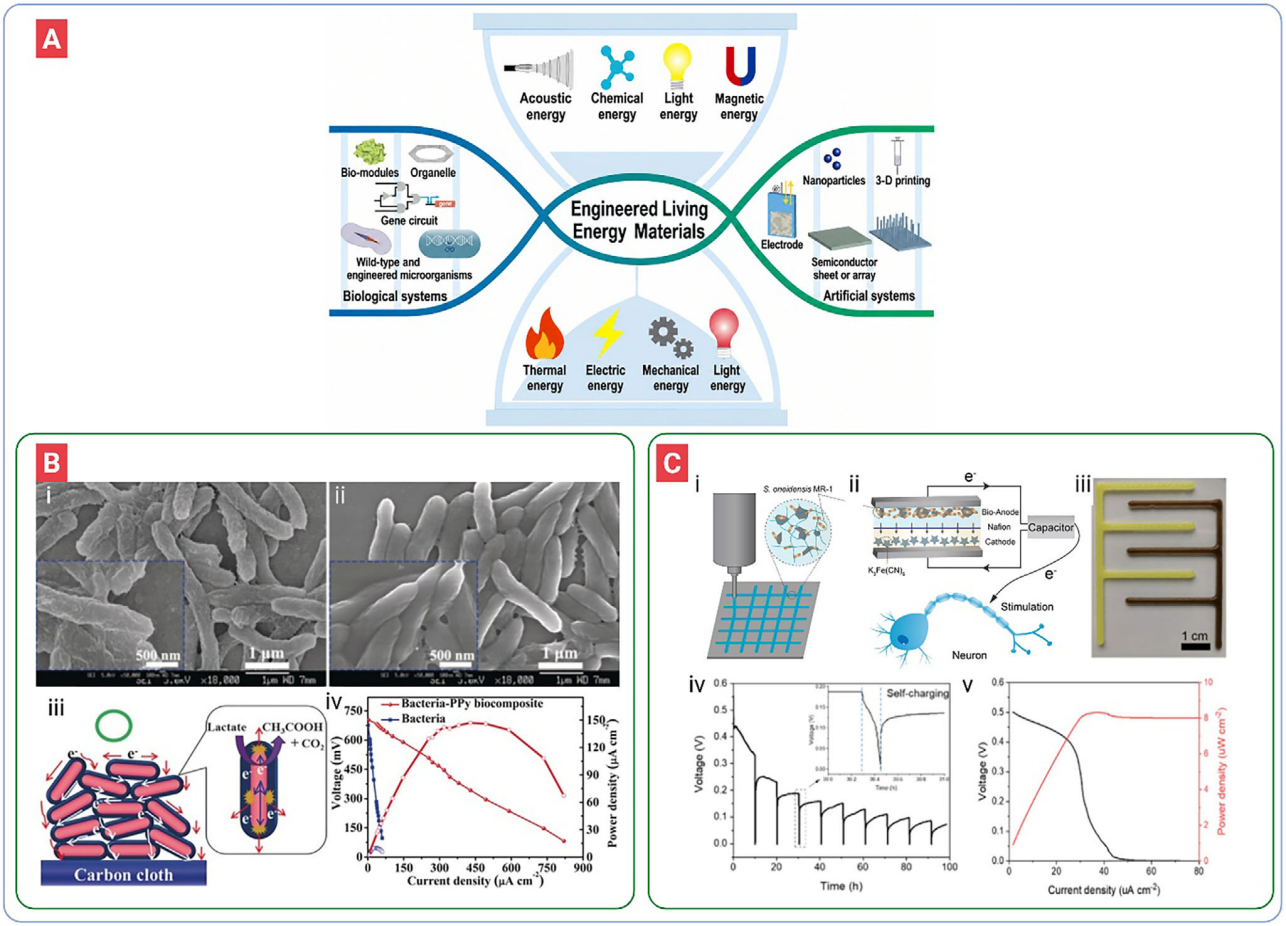
Engineered living materials for bioenergy applications. (A) Schematic illustration of engineered living energy materials integrating biological and artificial components to convert diverse energy inputs into useful energy outputs. Reproduced from Ref. [42], licensed under CC BY 4.0. Copyright. (B) Conductive polymer‐engineered *Shewanella oneidensis* MR‐1 for enhanced extracellular electron transfer in microbial fuel cells: SEM images of PPy‐coated *S. oneidensis* MR‐1 (i) and native *S. oneidensis* MR‐1 (ii), schematic of the direct‐contact electron transfer mechanism at a carbon‐cloth anode (iii), and polarization and power‐density curves comparing bacteria–PPy biohybrid and native bacterial electrodes (iv). Reproduced with permission from Ref. [43], Copyright 2017 Wiley‐VCH Verlag GmbH & Co. KGaA, Weinheim (C) Living hydrogel‐based bio‐battery containing *S. oneidensis* MR‐1 for neuromodulation: schematic of 3D bioprinting and bacterial encapsulation within an alginate hydrogel (i), bio‐battery‐powered neuron stimulation (ii), 3D‐printed living interdigital electrodes (iii), self‐charging performance over 100 h (iv), and polarization and power‐density characteristics of the bio‐battery (v). Reproduced with permission from [76], Copyright 2025 Wiley‐VCH GmbH.

Microbial fuel cells (MFCs) are a pressing application of living materials for renewable energy generation. In brief, these devices produce electricity via bacterial oxidation of organic substrates and transfer metabolic electrons to an electrode surface. Electron transfer occurs either via direct contact through outer membrane cytochromes and conductive pili or indirectly via redox mediators, which shuttle electrons between bacterial cells and the electrode surface. Electroactive microorganisms such as *Geobacter sulfurreducens* and *Shewanella oneidensis* are particularly attractive because of their highly efficient extracellular electron transfer pathways and ability to establish electroactive biofilms on conductive interfaces [74]. In addition to electricity generation, MFCs can simultaneously facilitate wastewater treatment, organic waste valorization, and recovery of valuable resources, thereby integrating energy production with environmental sustainability [74].

Similar to other living materials, MFCs face challenges related to efficiency, scalability, long‐term microbial viability, and operational stability. Their performance depends heavily on microbial metabolism, biofilm formation, electrode design, and extracellular electron transfer efficiency. Therefore, significant efforts have been directed toward developing conductive living interfaces that enhance electrochemical interactions between microorganisms and electrodes. The electron transfer rate for several electrode materials has been enhanced through incorporation of conducting polymers, including polypyrrole (PPy) and poly(3,4‐ethylenedioxythiophene) polystyrene sulfonate (PEDOT:PSS). Conductive polymers, for example, markedly enhanced electron transfer with *S. oneidensis* MR‐1 bacteria encapsulated in PPy by reducing charge‐transfer resistance nearly 20‐fold (Figure 2B) [43]. More recently, nanocomposite‐coated electrodes composed of conductive nanoparticles embedded within polymeric matrices have demonstrated substantial improvements in voltage generation, current density, and power density by promoting bacterial adhesion and facilitating extracellular electron transfer. For example, nanocomposite film‐coated zinc anodes significantly improved MFC performance, increasing power density up to 198 mW m^−^^2^ compared to uncoated systems [44]. These findings highlight the critical role of material engineering in regulating microbial‐electrode interactions and enhancing bioelectrochemical efficiency.

In addition to conductive polymers, carbon‐based living materials have emerged as promising electroactive scaffolds due to their high conductivity, mechanical strength, electrochemical stability, and biocompatibility. By integrating living cells with conductive carbon frameworks, these hybrid materials can achieve superior bioelectrochemical performance compared with traditional hydrogel‐based systems [75]. Importantly, the tunable electrical conductivity and electrochemical properties of carbon materials may enable self‐sensing and signaling capabilities within ELMs, thereby supporting autonomous electrochemical regulation and enhanced environmental adaptability [75]. In parallel, recent developments in engineered living composite materials further suggest that living biohybrid systems may eventually combine structural functionality with energy harvesting, environmental sensing, growth, and adaptive remodeling capabilities [12].

Beyond bioelectrochemical systems, cyanobacteria‐ and microalgae‐based living materials are gaining attention as sustainable platforms for solar‐driven bioenergy production [34, 70]. These biohybrid systems utilize the natural photosynthetic activity of living organisms to convert sunlight and carbon dioxide into bioelectricity or energy‐rich compounds under mild conditions. Advances in bioprinting and hydrogel engineering have further improved their structural organization, metabolic stability, and long‐term functionality. For instance, 3D‐printable living hydrogels containing conductive biofilms encapsulated within alginate matrices have recently been developed as portable bioenergy devices capable of self‐charging electrochemical performance while maintaining cell viability above 90% (Figure 2C) [76]. Compared with MFCs, which rely on microbial oxidation of organic substrates and efficient electron transfer to electrodes, photosynthetic living systems can directly harness light and CO_2_ but are more strongly constrained by illumination, mass transport, and photosynthetic efficiency [42]. These systems may support applications in portable electronics, implantable bioelectronics, biosensing, and sustainable energy technologies.

The gap between current MFC performance (typically 0.1–10 mW m^−3^ in practical systems) and the power densities needed for commercial viability remains substantial, and it reflects a genuinely difficult engineering problem: biological metabolism is inherently variable, and optimizing electrode architecture, biofilm adhesion, and electron‐transfer efficiency simultaneously across a system that continues to evolve biologically is not straightforward [45]. Living cells contribute not only to energy generation but also to the formation and functional regulation of the material itself, an advantage that distinguishes these systems from conventional electrochemical cells but also introduces biological complexity that complicates performance prediction [77]. Furthermore, synergistic integration of synthetic biology, conductive materials, semiconductors, additive manufacturing, and bioinspired hybrid architectures may enable future living energy systems with enhanced programmability, environmental resilience, and adaptive electrochemical functionality [56, 73].

The path toward commercially viable living bioenergy systems depends on addressing key bottlenecks through the combined efforts of synthetic biology and materials engineering. Oxygen sensitivity remains a major challenge, as many highly electroactive bacteria are strict anaerobes, while maintaining oxygen‐free conditions at scale can be expensive and technically demanding. Biofilm management presents another challenge, since the electroactive biofilms that drive electricity generation can also gradually foul electrode surfaces and reduce performance over time. Strategies that combine genetic control of biofilm growth and composition with conductive electrode architectures resistant to fouling may therefore provide a promising route toward improving the long‐term performance of these systems.

### 2.5 Biomedicine and Wearable Systems

Biomedical applications place the most demanding constraints on living materials of any domain in this review. The material must perform reliably within a living host that actively works to eliminate it: immune cells attack bacterial components, fibrotic encapsulation isolates implanted devices, and the physiological environment degrades most scaffolds over timescales far shorter than clinical utility would require. Yet this same physiological complexity is precisely what living materials are positioned to address. ELMs can sense local biochemical signals, produce therapeutic molecules on demand, and in some cases remodel their scaffold properties in response to tissue‐level cues [77]. In comparison to conventional passive biomaterials, living materials contain metabolically active biological components that can sense, respond to, and interact with physiological environments [78]. This enables adaptive therapeutic functions, self‐regulation, continuous bioactive molecule production, and environmentally responsive behavior beyond the capabilities of traditional smart materials. Advances in synthetic biology, biofabrication, and soft bioelectronics have further promoted the development of multifunctional biohybrid systems that integrate living cells with responsive and conductive scaffolds for dynamic biomedical applications [79]. Across these diverse applications, the central design challenges are largely shared: maintaining cell viability and functional stability, enabling controlled communication with the physiological environment, and ensuring immune compatibility and biological containment.

In drug delivery and regenerative medicine, ELMs have increasingly been explored as localized therapeutic systems capable of sustained biological activity and environmentally responsive treatment [80, 81]. In this direction, hyaluronic acid–based hydrogels containing *Lactobacillus reuteri* have been used to promote healing of infected wounds by secreting lactic acid and antimicrobial agents, thereby reducing inflammation, enhancing collagen deposition, and accelerating tissue repair (Figure 3A) [82]. More broadly, engineered probiotics and bacteria‐based living therapeutics are emerging as promising platforms for treatment of inflammatory diseases, cancer, and metabolic disorders through programmable secretion of therapeutic compounds, modulation of local microbiota composition, and spatiotemporally controlled therapeutic activity [83]. In particular, recent developments in probiotics delivery have demonstrated how biomaterial coatings based on alginate, chitosan, lipid membranes, and responsive hydrogels can substantially improve microbial survival, therapeutic retention, and colonization under harsh physiological conditions. These protective matrices shield microorganisms against gastric acid, bile salts, oxidative stress, and immune‐mediated damage while enabling targeted adhesion and controlled therapeutic release [84]. Furthermore, engineered coatings may provide additional functionalities such as reactive oxygen species scavenging, inflammation‐responsive activation, and modulation of pathological microenvironments, thereby improving therapeutic performance in inflammatory bowel disease, colorectal cancer, and chronic inflammatory disorders [84].

**FIGURE 3:**
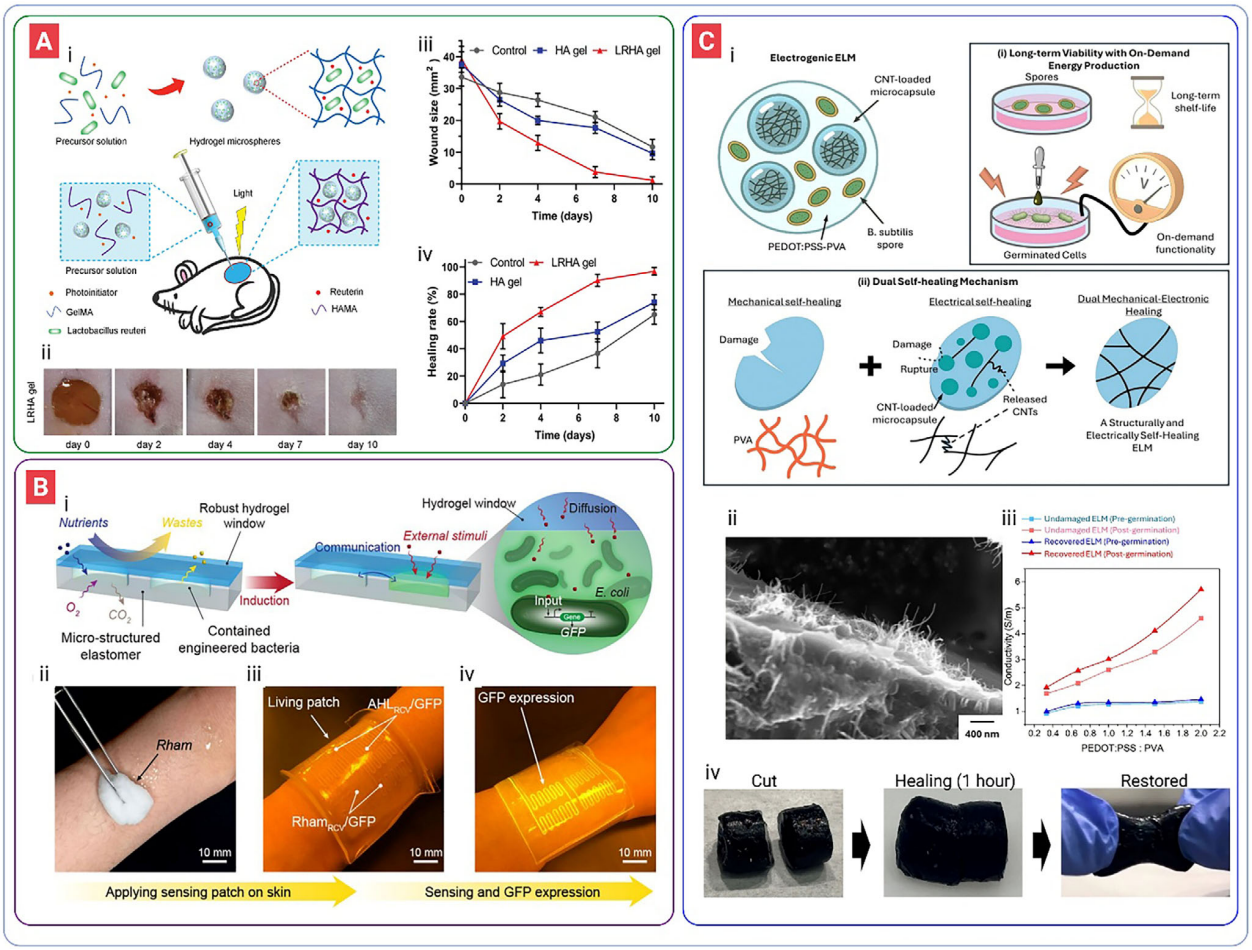
Representative applications of engineered living materials (ELMs) in biomedicine and wearable bioelectronics. (A) Living probiotic hydrogel for infected wound healing. (i) Schematic illustration of the fabrication of Lactobacillus reuteri‐loaded hydrogel microspheres and their incorporation into a hyaluronic acid methacrylate (HAMA)‐based hydrogel through in situ photocrosslinking. (ii) Representative images of wound closure during treatment. (iii, iv) Changes in wound size and healing rate in mice treated with the living hydrogel compared with control groups. Reproduced from Ref. [82], licensed under CC BY 4.0. Copyright. (B) Stretchable living material for wearable biosensing. (i) Schematic of a hydrogel–elastomer hybrid material containing genetically engineered Escherichia coli, where the hydrogel supplies nutrients, water, and oxygen while enabling molecular communication with the environment. (ii–iv) Demonstration of a skin‐mounted living sensor patch, where exposure to rhamnose induces GFP expression in engineered bacterial cells, generating a visible fluorescent response. Reproduced with permission from [48], Copyright 2017 National Academy of Sciences (C) Self‐healing electrogenic living hydrogel for durable bioelectronics. (i) Conceptual design of a PEDOT:PSS–PVA hydrogel containing CNT‐loaded microcapsules and Bacillus subtilis spores, enabling long‐term viability, on‐demand activation, and dual self‐healing functionality. (ii) SEM image showing CNT release from ruptured microcapsules at a damaged region. (iii) Conductivity measurements of undamaged and self‐healed ELMs before and after spore germination. (iv) Demonstration of mechanical self‐healing following cutting and subsequent restoration of stretchability. Reproduced with permission from [93], Copyright 2025 American Chemical Society.

ELMs have also shown significant promise in biosensing and wearable diagnostic systems because living cells can function as responsive sensing elements capable of detecting biochemical, mechanical, and environmental stimuli with high sensitivity and specificity. For example, a glove‐based sensor and wearable skin patch were developed by encapsulating engineered *E. coli* within a hydrogel–elastomer hybrid composed of polyacrylamide (PAAm)‐alginate hydrogel and silicone elastomer to detect multiple chemical targets such as rhamnose, N‐acyl homoserine lactone, and anhydrotetracycline through fluorescence‐based responses (Figure 3B) [48]. In this system, the scaffold maintained nutrient transport, preserved cell viability during mechanical deformation, and facilitated cell–environment communication while retaining flexibility and air permeability. Similarly, engineered microbial biosensors and genetically programmed bacteria have been developed for real‐time monitoring of disease biomarkers, inflammation‐associated metabolites, DNA sequences, and protease activity [49, 85]. Unlike conventional electronic sensors, these biologically integrated systems can actively process environmental information through genetic circuits and metabolic signaling pathways, thereby enabling adaptive sensing, signal amplification, and programmable diagnostic responses.

The convergence of living materials with flexible bioelectronics has further expanded the scope of wearable healthcare technologies and human–machine biointerfaces (Cyborganics) [86, 87]. Recent advances in electrically conductive living hydrogels, electrogenic bacterial systems, and soft bioelectronic interfaces have enabled fabrication of flexible devices capable of physiological monitoring, adaptive signal transduction, self‐powered sensing, and bioenergy harvesting [51]. Conductive polymer‐based living systems utilizing PEDOT:PSS and electroactive microorganisms have demonstrated improved electrical conductivity, stretchability, and bioelectronic responsiveness under dynamic physiological conditions [88]. In parallel, wearable biofuel‐cell systems integrating enzymatic or microbial catalytic platforms have enabled continuous metabolite sensing and localized energy generation in wearable formats [89]. Compared with conventional wearable electronics, living bioelectronic systems possess the unique ability to dynamically regenerate functionality, adapt to environmental stimuli, and maintain biologically interactive behavior over time. Consequently, these systems are increasingly viewed as promising candidates for next‐generation adaptive healthcare devices and self‐sustaining biomedical interfaces [90].

Another rapidly emerging direction involves tissue‐integrated and adaptive biointerfaces, where living materials establish long‐term functional interactions with biological tissues. Advances in soft neural interfaces, organoids, biointegrated electronics, and extracellular matrix‐inspired living scaffolds have enabled the development of dynamic interfaces capable of supporting neural communication, regenerative remodeling, inflammation monitoring, and adaptive tissue integration [91, 92]. For example, bacteria‐integrated hydrogel electronics have been developed to simultaneously manage inflammatory responses and enable electrophysiological monitoring through biointegrated living electronic systems [16]. In addition, self‐healing electrogenic hydrogels and adaptive conductive composites capable of restoring electrical conductivity following mechanical damage have demonstrated improved durability and operational stability under physiologically relevant conditions (Figure 3C) [93]. Collectively, these applications converge on a common design principle: the living component must maintain biological activity while continuously communicating with the surrounding tissue, whereas the scaffold must simultaneously provide protection, transport, mechanical support, and functional integration.

Despite these promising advances, several major translational barriers continue to limit the clinical implementation of living biomedical materials. One of the most critical challenges is the host immune response toward living microorganisms and bacterial components, particularly lipopolysaccharides (LPS) from gram‐negative bacteria, which can induce strong inflammatory reactions, cytokine activation, and chronic immune responses. In addition, bacterial translocation, uncontrolled microbial proliferation, genetic instability, biofilm overgrowth, and unintended environmental release of engineered organisms remain significant biosafety concerns. Implantable and tissue‐integrated living systems may further suffer from fibrosis, foreign‐body responses, vascularization limitations, degradation of bioelectronic interfaces, and long‐term instability under physiological conditions. To mitigate these limitations, recent strategies have focused on the development of biocompatible and immunomodulatory matrices such as polyethylene glycol (PEG), alginate, and bioinert hydrogels together with genetically engineered microorganisms exhibiting reduced immunogenicity and improved containment mechanisms [94]. Furthermore, advanced encapsulation approaches including multilayer coatings, microfluidic biofabrication, and stimuli‐responsive hydrogels may improve microbial protection while minimizing unintended host interactions [84].

The immune response deserves particular emphasis as a defining translational barrier for biomedical ELMs, a challenge that is less prominent in most other application domains. Progress here will likely depend less on materials innovation alone and more on combinations of immune‐compatible scaffold chemistry, genetic attenuation of immunogenic bacterial surface components, and cell‐specific containment strategies that allow therapeutic function while limiting systemic exposure. Without credible solutions to immune compatibility and long‐term biocontainment, further advances in synthetic biology, immune engineering, biofabrication, and long‐term safety assessment will be essential before living materials can achieve widespread clinical translation.

## 3 Self‐Maintainable and Autonomous Living Materials

The systems described in Section 2 are largely reactive: they respond to a stimulus—a crack, a pollutant, an organic substrate—and produce a single, predefined output. What they cannot yet do is what all living organisms do as a matter of course: monitor their own state, regulate multiple competing processes simultaneously, allocate resources between survival and function, and sustain that behavior indefinitely without external intervention. Closing this gap is the defining engineering challenge for the next generation of ELMs. Across these application domains, the key distinction is therefore not the biological component or intended application, but the degree of functional autonomy achieved. We propose that ELMs can be viewed along a functional progression from responsive systems, through self‐maintainable materials, toward autonomous systems capable of integrated sensing, feedback regulation, and sustained operation with minimal external intervention.

Early‐generation living materials primarily relied on isolated functionalities such as stimulus responsiveness or self‐healing; next‐generation systems increasingly aim to integrate autonomous sensing, adaptive feedback regulation, dynamic remodeling, and energy management within a single platform [7, 19]. The key distinction therefore lies in the level of functional integration: responsive systems produce predefined outputs to specific stimuli, self‐maintainable systems use these responses to preserve functionality, and autonomous systems further integrate sensing and feedback to regulate their behavior with minimal external intervention. Unlike conventional smart materials, which generally operate through predefined physicochemical responses, autonomous living materials integrate metabolically active cells capable of continuously interacting with their environment through feedback‐controlled biological processes. Consequently, these systems can dynamically regulate their structural and functional properties over time, enabling a transition from static materials toward adaptive matter with life‐inspired behaviors [77]. This progression from passive materials to autonomous living systems, together with the associated levels of autonomy, enabling technologies, translational barriers, and future development roadmap, is summarized in Figure 4.

**FIGURE 4:**
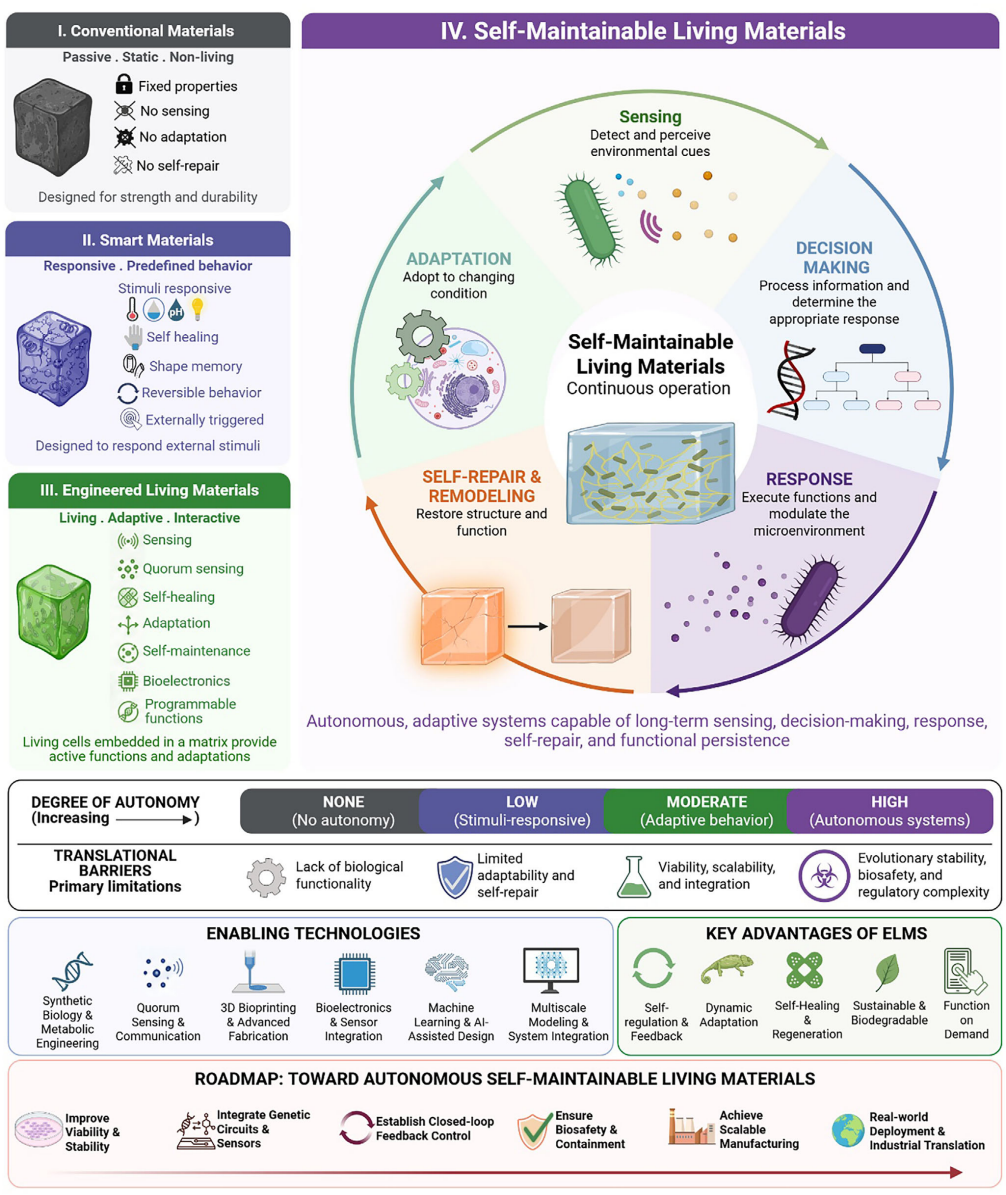
Evolution of material systems toward autonomous self‐maintainable living materials. Conventional materials, smart materials, engineered living materials (ELMs), and autonomous living systems are compared based on increasing autonomy and functionality. Autonomous systems are envisioned to operate through closed‐loop sensing, decision‐making, response, and adaptation. The figure also summarizes major translational barriers, enabling technologies, key advantages of ELMs, and a roadmap for future development.

Self‐maintainable materials are systems capable of preserving their functionality through biological repair, adaptation, and self‐regulation. These systems display self‐healing capacity and, similar to living biological tissues, can simultaneously sense, respond, adapt, and reorganize under environmental stress. Such systems may maintain their functionality for prolonged periods without external repair or reconfiguration [95]. In biological systems, self‐maintenance is achieved through tightly regulated feedback loops involving sensing, signaling, repair, and remodeling pathways. Inspired by these mechanisms, recent engineered living materials have demonstrated the ability to initiate intracellular signaling cascades, release bioactive molecules, regulate local microenvironments, and modulate cellular responses under specific stimuli. These responses may induce cell migration, proliferation, differentiation, or dynamic modification of scaffold properties to improve adaptation to changing conditions. In particular, autonomous living scaffolds inspired by the osteogenic microenvironment have been proposed for bone regeneration, where sustained release of living cells, coupled with dynamic regulation of osteogenesis and angiogenesis, enables long‐term remodeling and regenerative adaptation [96]. The integration of this type of living material in implants and prosthetic devices can revolutionize healthcare by allowing the monitoring of physiological conditions and providing on‐demand responses [96]. However, achieving true autonomy presents some difficult challenges, such as integrating multiple self‐maintaining functions and addressing biocompatibility to prevent uncontrolled growth or immune responses [5]. Such systems demonstrate how living materials may actively participate in tissue regeneration rather than serving solely as passive structural supports.

Autonomous living materials extend self‐maintainable systems by integrating continuous sensing, closed‐loop feedback, and adaptive responses with minimal external intervention. Continuous monitoring of environmental conditions enables these systems to detect external stimuli and dynamically regulate downstream responses, thereby creating adaptive feedback‐controlled architectures analogous to biological homeostasis [97]. Functional closed‐loop systems generally require integrated sensing and response modules, both of which may be cell‐based, material‐based, or biohybrid combinations of the two [11]. Through these interactions, living materials can process environmental information, adapt their behavior, and regulate material performance in real time. Such integrated biofeedback systems enable living materials to self‐optimize and dynamically maintain physiological compatibility, representing a key requirement for true material autonomy. For example, after initiating self‐healing responses, embedded cells may downregulate metabolic activity to conserve energy or activate stress‐protective pathways during prolonged environmental exposure, thereby improving long‐term material stability and survival [7].

Intelligent stimuli‐responsive mechanisms are therefore essential for the development of self‐maintainable living materials [98]. In one example, engineers integrated cyanobacteria into thermoresponsive hydrogels capable of dynamically altering scaffold properties through secretion of extracellular enzymes in response to environmental changes [99]. These findings demonstrate how intrinsic biological activity can be harnessed to create environmentally adaptive materials that autonomously regulate their own structural and functional states. Similarly, programmable bacterial biofilms have emerged as highly promising platforms for autonomous ELMs because biofilms naturally possess self‐organizing, self‐healing, and environmentally responsive characteristics [100]. Through synthetic biology approaches, bacterial biofilms can be engineered to incorporate genetic circuits enabling environmental sensing, dynamic patterning, self‐repair, and programmable functional outputs. Such systems increasingly blur the distinction between materials and living systems by introducing active adaptation and biologically mediated decision‐making into the material itself.

Recent developments have further expanded autonomous functionality through integration of living systems with advanced materials engineering, biofabrication, and electronic interfaces. ELMs can now be fabricated using 3D bioprinting, electrospinning, living assembly, and hybrid scaffold engineering approaches that enable precise spatial organization of cells and material components [101]. These fabrication strategies allow integration of multiple functional modules within a single material platform, including structural reinforcement, sensing, actuation, and therapeutic activity. Importantly, advanced fabrication techniques also improve nutrient diffusion, cellular viability, mechanical robustness, and environmental responsiveness, which are all essential for long‐term self‐maintenance [101].

Concepts from adaptive and self‐maintainable electronics may also inform the development of autonomous living materials. For example, graphene‐PEDOT:PSS‐based electronic materials with skin‐like characteristics have recently demonstrated simultaneous self‐healing, multiplex sensing, thermal responsiveness, flexibility, and adaptive environmental interactions within a single cohesive platform [95]. Unlike conventional self‐healing electronic materials that only restore mechanical integrity after damage, these systems can additionally sense, respond, and adapt to environmental conditions in a manner resembling biological tissues. Such multifunctional materials may ultimately enable autonomous soft robots, adaptive prosthetics, wearable bioelectronics, and self‐maintaining biomedical implants capable of prolonged operation under dynamic physiological conditions [95].

Another important direction involves the development of durable living materials capable of maintaining dormant functionality under harsh environmental conditions. One major challenge limiting practical implementation of ELMs is the relatively short lifespan and environmental sensitivity of living cells. To address this limitation, recent studies have utilized bacterial spores embedded within bacterial cellulose matrices to create resistant living materials with programmable on‐demand functionality [102]. In these systems, dormant spores remain metabolically inactive yet viable under stressful conditions and can later germinate to restore biological functionality when exposed to appropriate stimuli. Thus, such strategies substantially improve long‐term storage stability, environmental tolerance, and operational durability while preserving the adaptive advantages of living systems. These approaches may prove especially important for future deployment of autonomous living materials outside controlled laboratory environments.

Beyond self‐healing and environmental responsiveness, emerging perspectives increasingly envision autonomous living materials as dynamic systems capable of memory, learning, and collective adaptation. Materials inspired by living functions have recently incorporated concepts such as feedback‐controlled homeostasis, associative learning, dissipative self‐organization, and adaptive non‐equilibrium behavior [103]. These developments suggest that future living materials may eventually exhibit increasingly sophisticated behaviors resembling rudimentary biological intelligence. In parallel, integration of artificial intelligence (AI) with living materials has been proposed as a route toward adaptive hybrid systems capable of autonomous decision‐making, multifunctional environmental interactions, and complex self‐regulation [104]. Although these concepts remain largely exploratory, they highlight the possibility that future living materials may evolve from responsive matter toward highly integrated adaptive systems operating at the interface of biology, materials science, and computational intelligence.

Despite these remarkable advances, achieving true autonomy in living materials remains highly challenging. Current systems still face major limitations related to metabolic burden, energy supply, long‐term cellular viability, evolutionary instability, manufacturing scalability, and reliable integration of multiple self‐regulating functions within a single platform [19]. Additional concerns involve biosafety, uncontrolled cellular proliferation, environmental release of engineered organisms, and host immune responses in biomedical applications. Furthermore, maintaining stable performance under fluctuating environmental conditions while preserving predictable biological behavior remains difficult due to the inherent variability and complexity of living systems [7]. Consequently, future progress in autonomous living materials will likely depend on advances in synthetic biology, systems biology, biofabrication, immune engineering, adaptive electronics, and artificial intelligence‐assisted control systems.

Overall, autonomous and self‐maintainable living materials represent a major step toward the development of adaptive material systems capable of continuous interaction with their environment. Consequently, by integrating sensing, response, self‐healing, remodeling, and feedback‐controlled regulation into unified platforms, these systems may eventually enable long‐lasting biomedical devices, intelligent infrastructure, self‐sustaining environmental technologies, and adaptive soft robotic systems. Nevertheless, substantial scientific and translational challenges remain before fully autonomous living materials can achieve widespread real‐world implementation.

## 4 Challenges and Opportunities

Despite remarkable advances in ELMs, several major scientific, technological, and translational challenges continue to limit their widespread implementation. Unlike most conventional smart materials, living materials essentially rely on metabolically active biological systems that continuously consume nutrients, generate waste products, and alter their local chemical environment as a direct consequence of being alive. This biological activity is the source of their capability, but also of their fragility: long‐term performance depends on maintaining adequate nutrient diffusion, oxygen transport, and waste removal within a scaffold architecture that must simultaneously preserve mechanical integrity. In many cases, engineered cells gradually lose their programmed functionality because of mutation accumulation, phenotype drift, evolutionary selection pressure, or metabolic competition between survival and the engineered function. Furthermore, balancing cellular proliferation with structural stability remains difficult, as uncontrolled growth may compromise material integrity, while excessive confinement may suppress the cellular activity the material depends on. These limitations become particularly acute in autonomous living systems, where sustained operation requires continuous coordination between sensing, response, repair, and energy‐management processes [56].

As discussed earlier, maintaining the stability and durability of living materials under real‐world conditions remains a major challenge. The diverse environmental and mechanical stresses encountered outside controlled laboratory settings can significantly affect cell viability and function, making it difficult to achieve predictable and reliable biological performance over extended periods. In addition, many living materials still depend on highly hydrated environments such as hydrogels, which may limit long‐term mechanical robustness, environmental tolerance, and scalability. Although significant progress has been made in improving cell embedding and survival through responsive polymers, conductive matrices, nanocomposites, and advanced hydrogel systems, further development of mechanically resilient yet biologically permissive materials remains essential. Emerging fabrication technologies including 3D bioprinting, electrospinning, and hierarchical biofabrication may provide important opportunities for improving spatial organization, nutrient transport, mechanical integrity, and long‐term system stability [101].

Translational and biosafety considerations also represent major barriers to practical implementation of living materials. Unlike abiotic smart materials, living systems introduce unique biological risks including uncontrolled cellular proliferation, horizontal gene transfer, ecological disruption, and unintended environmental release of engineered organisms. The relative importance of these risks, however, depends strongly on the application. Environmental and agricultural ELMs face greater concerns related to environmental release, horizontal gene transfer, and ecological interactions, whereas biomedical ELMs are more strongly constrained by immune compatibility, microbial containment, and host safety. In construction applications, long‐term biological stability and unintended environmental release during material degradation or disposal may represent more relevant concerns. In biomedical applications, host immune responses toward microbial components, particularly LPS, may induce inflammation, fibrosis, cytokine activation, and chronic immune reactions that compromise long‐term functionality and clinical safety. Additionally, maintaining reliable containment of engineered microorganisms while preserving therapeutic or adaptive functionality remains highly challenging. Regulatory uncertainty further complicates commercialization because living materials do not fit easily within existing frameworks developed for either biomaterials or biologics. Standardization, reproducibility, long‐term storage, sterilization compatibility, and large‐scale manufacturing also remain insufficiently developed for widespread industrial adoption. Consequently, future progress in the field will require close integration between synthetic biology, immune engineering, regulatory science, systems biology, and advanced manufacturing technologies.

Beyond technical and regulatory challenges, societal acceptance will play a critical role in determining the future implementation of engineered living materials. Unlike conventional materials, ELMs incorporate living organisms that may continue to interact with their environment after deployment, raising concerns regarding environmental release, long‐term ecological impacts, and public trust. These concerns may be particularly significant in applications involving open environmental systems, agricultural settings, and biomedical devices containing engineered microorganisms. Public perception of genetically modified organisms has historically influenced the adoption of biotechnology‐based innovations, highlighting the importance of transparent risk assessment, stakeholder engagement, and responsible communication. Consequently, successful translation of ELMs will require not only advances in materials science and synthetic biology but also the development of governance frameworks that address ethical considerations, environmental stewardship, societal expectations, and long‐term monitoring of deployed systems.

Evolutionary stability remains particularly underexplored during long‐term deployment. Because the living components of ELMs continuously replicate and adapt under environmental selection pressures, engineered functions may gradually become unstable or be lost over time. The challenge becomes even more pronounced in autonomous systems where sensing, decision‐making, and response mechanisms rely on complex synthetic gene circuits. Future research should therefore focus on developing evolutionarily robust genetic architectures, self‐correcting regulatory networks, and biocontainment strategies that preserve functionality while minimizing unintended adaptation. Advances in synthetic biology, including genome stabilization technologies, programmable kill‐switches, and feedback‐regulated control systems, may provide important tools for maintaining predictable long‐term behavior. Ultimately, achieving reliable evolutionary stability will be essential for transforming engineered living materials from laboratory demonstrations into durable real‐world technologies.

At the same time, rapid developments in synthetic biology are creating unprecedented opportunities for engineering increasingly adaptive and programmable living systems. Advances in metabolic engineering, synthetic gene circuits, quorum sensing regulation, and multicellular communication networks now enable increasingly precise control over cellular behavior, environmental responsiveness, and functional outputs. Through rational genetic programming, living cells can potentially be engineered to dynamically regulate growth, energy consumption, self‐healing, therapeutic activity, and environmental adaptation in response to changing external conditions. Recent progress in programmable bacterial biofilms, engineered microbial consortia, and biologically grown living composites further demonstrates the possibility of constructing materials capable of self‐organization, environmental sensing, and autonomous functional regulation [100]. In microbial consortia, division of metabolic labor and quorum‐sensing‐mediated communication can distribute complex functions across different populations, reducing the functional burden on individual strains. However, competition for nutrients and space may alter community composition over time, potentially disrupting the intended division of labor and reducing functional stability. Controlling the spatial organization and relative abundance of different populations will therefore be important for maintaining predictable long‐term performance in multi‐microbial ELMs [7, 105]. Moreover, development of dormant yet activatable systems such as spore‐integrated living materials may substantially improve long‐term storage stability and environmental robustness while preserving programmable biological functionality [102, 106].

## 5 Outlook

Beyond synthetic biology, convergence of living materials with bioelectronics, AI, robotics, and computational modeling may fundamentally reshape the future of adaptive material systems. Computational approaches, including systems biology simulations, digital twins, and AI‐assisted biomaterial design, may improve prediction of cell–material interactions, optimize material architectures, and enable real‐time adaptive control of living systems under dynamic conditions. Integration of bioelectronic interfaces with living materials may further support closed‐loop sensing, feedback regulation, and adaptive responses analogous to biological homeostasis. In parallel, advances in soft bioelectronics and self‐maintainable electronic materials are enabling development of multifunctional systems capable of simultaneously sensing, responding, self‐healing, and adapting to environmental stimuli in a manner increasingly resembling biological tissues [95]. These developments suggest that future living materials may evolve beyond passive responsive platforms toward highly integrated adaptive matter capable of dynamic interaction with complex environments.

The deeper question facing the field is not whether living materials can perform individual functions—the evidence reviewed here makes clear that they can—but whether they can do so reliably, safely, and economically at scales that matter. Answering that question requires more than incremental performance improvements. It requires confronting the governance gap: living materials do not fit neatly within regulatory frameworks designed for either biomaterials or biologics, and no international standards currently exist for their safety assessment, environmental containment, or long‐term monitoring. Building those frameworks in parallel with technical development, rather than after it, will be essential if the field is to move beyond demonstration studies. Autonomous living materials may eventually integrate distributed sensing, collective decision‐making, adaptive remodeling, and biologically mediated feedback regulation within a single platform. Emerging perspectives even suggest the possibility of combining living materials with artificial intelligence and neural‐network‐assisted control systems to generate increasingly sophisticated hybrid systems capable of autonomous environmental interaction and multifunctional behavior [104]. Although such concepts remain largely exploratory, they highlight the growing convergence between materials science, synthetic biology, bioelectronics, and computational intelligence.

Although significant progress has been achieved in developing responsive and multifunctional living materials, the realization of fully autonomous self‐maintainable systems will require advances across several interconnected dimensions. Current ELMs primarily exhibit isolated capabilities such as sensing, self‐healing, or metabolite production, whereas future generations must integrate these functions within closed‐loop architectures capable of continuous environmental monitoring, adaptive decision‐making, and long‐term self‐regulation. Progress toward autonomy should therefore be evaluated not only by the number of integrated functions, but also by their response accuracy, feedback stability, duration of autonomous operation, and dependence on external intervention. A practical roadmap toward this goal involves several interconnected milestones, including (i) improving long‐term cellular viability and metabolic stability, (ii) integrating sensing and synthetic regulatory circuits, (iii) establishing reliable closed‐loop feedback and adaptive behavior, (iv) ensuring robust genetic containment and biosafety, and (v) developing scalable manufacturing platforms capable of preserving biological functionality during production and deployment (Figure 4). Achieving these objectives will require close collaboration among materials scientists, synthetic biologists, bioengineers, computational modelers, and regulatory authorities. As these barriers are progressively overcome, living materials may evolve from reactive biohybrid systems into truly autonomous technologies capable of maintaining functionality, adapting to changing environments, and operating with minimal human intervention over extended periods.

Ultimately, the long‐term significance of ELMs may extend far beyond the development of self‐healing or stimuli‐responsive systems. Inspired by the resilience, adaptability, and dynamic behavior of natural organisms, future living materials may become active participants within their environments, functioning in ways akin to living biological systems rather than passive structural components. Such systems could potentially support intelligent biomedical implants, adaptive infrastructure, self‐sustaining environmental technologies, autonomous soft robotics, and multifunctional biohybrid ecosystems capable of continuous interaction with complex surroundings. Nevertheless, achieving this vision will require overcoming substantial scientific, ethical, regulatory, and technological challenges. The ultimate challenge is therefore no longer simply engineering materials that respond to stimuli but constructing adaptive material systems capable of sustained autonomous interaction with real‐world environments while maintaining predictable, safe, and controllable behavior over prolonged periods of operation.

## 6 Conclusion

ELMs are redefining the boundaries between biology and materials science by integrating metabolically active organisms within functional material architectures. More importantly, they represent a transition from passive and stimuli‐responsive materials toward systems capable of sensing, adaptation, self‐maintenance, and increasingly autonomous behavior. While significant challenges related to long‐term stability, biosafety, scalability, and regulatory implementation remain, rapid advances in synthetic biology, biofabrication, and systems engineering are steadily expanding the capabilities of these platforms. Realizing truly autonomous ELMs, however, will require these functions to operate reliably within integrated closed‐loop systems while maintaining predictable and safe behavior over extended periods. As these barriers are overcome, living materials may evolve into adaptive systems capable of continuous interaction with complex environments, opening new opportunities in healthcare, infrastructure, environmental management, and energy technologies. The continued convergence of biology and materials science therefore holds the potential to establish a new generation of intelligent and self‐maintainable materials inspired by the fundamental principles of living systems.

## Author Contributions

**Mohammadsadegh Nadimifar**: writing – original draft, conceptualization, visualization, investigation. **Tatiane Eufrásio‐da‐silva**: writing – review and editing, visualization. **Lidia Maeso**: writing – review and editing, visualization. **Gorka Orive**: conceptualization, writing – review and editing, funding acquisition. **Alireza Dolatshahi‐pirouz**: conceptualization, funding acquisition, writing – review and editing.

## Conflicts of Interest

The authors declare no conflicts of interest.

## Data Availability

Data sharing not applicable to this article as no datasets were generated or analysed during the current study.
